# Matched Fruit–Larva Metabolomics Identifies Host-Associated Metabolic Signatures After Multi-Generational Laboratory Acclimation in *Zeugodacus tau* (Tephritidae: Diptera)

**DOI:** 10.3390/insects17090962

**Published:** 2026-09-16

**Authors:** Wei Shi, Ruixiang Li, Rui Sun, Jun Cao

**Affiliations:** 1 Yunnan Key Laboratory of Biological Adaptation, Conservation and Utilization, School of Ecology and Environmental Science, Yunnan University, Kunming 650091, China; 2 State Key Laboratory of Vegetation Structure, Function and Construction (VegLab), School of Ecology and Environmental Science, Yunnan University, Kunming 650091, China

**Keywords:** *Zeugodacus tau*, host shift, matched fruit–larva metabolomics, metabolic response, biomarker

## Abstract

*Zeugodacus tau* (Walker) is a polyphagous invasive tephritid capable of cross-family host shifts from ancestral Cucurbitaceae crops to phylogenetically divergent commercial fruits, causing severe horticultural economic losses. As an obligate fruit-boring pest, *Z. tau* largely relies on larval metabolic plasticity to achieve successful colonization of novel hosts during host shifts. However, few matched fruit–larva metabolomic studies integrate host chemistry to larval physiology, hindering sustainable pest control development. We performed untargeted LC–MS metabolomics on matched fruit and third-instar larval samples from eight hosts after multi-generational acclimation. Principal component analysis (PCA) split fruit and larval metabolomes into cucurbit and non-cucurbit clusters, indicating that larval metabolic profiles align with host fruit chemistry during host shifts. Cucurbit fruits contained uniformly high γ-aminobutyric acid (GABA), and their larvae exhibited enriched glutamate (Glu)-centred amino acid pathways. Non-cucurbit fruits showed heterogeneous chemical composition: larvae-fed banana, mango and pitaya all possessed high UDP-glucose (UDPG), whose levels correlated with fruit glucose-6-phosphate (G6P) enriched in banana and mango. Overall, larvae colonizing distinct non-cucurbit hosts deployed a suite of divergent carbohydrate metabolic modules to respond to variable fruit chemical microenvironments. Banana and pitaya larvae showed enhanced starch–sucrose turnover, whereas mango larvae featured prominent ascorbate and aldarate metabolism; both pathways are closely connected to carbohydrate homeostasis. Orange and guava larvae activated distinct gluconeogenic branches. Gluconeogenesis itself constitutes an important component of carbohydrate metabolism. This dual-metabolome profiling uncovered host-specific biomarkers and two divergent metabolic strategies underlying the host shift in *Z. tau*, characterizing host-linked metabolic variation after long-term laboratory acclimation, which delivers correlative biochemical clues for subsequent pest management research.

## Simple Summary

*Zeugodacus tau* (Walker), a destructive invasive fruit fly that originally fed on cucurbits, readily infests commercial fruits and triggers heavy horticultural losses. Larval tolerance to fruit chemical microenvironments largely determines host-shift success, yet host–larva matched metabolomics has not been characterized for this pest. Here we generated the first matched dual metabolome dataset of multi-generation acclimated *Z. tau* and eight host fruits. Larval fitness was far higher on ancestral cucurbits than novel fleshy fruits. We identified two distinct sets of metabolic signatures correlated with host type: larvae reared on cucurbit hosts show enrichment of amino acid metabolism pathways, whereas larvae feeding on fleshy fruits display altered carbohydrate metabolic profiles. The candidate metabolite biomarkers identified herein provide correlative clues that could inform follow-up research on pest surveillance and eco-friendly targeted control tactics, pending further functional and field validation.

## 1. Introduction

Tephritid fruit flies constitute a group of destructive invasive pests that threaten horticultural production, food security and global agricultural trade across tropical and subtropical regions [1]. As polyphagous herbivores, they readily shift onto novel host plants, aggravating worldwide crop yield losses [2,3,4]. Dissecting the adaptive traits that drive host range expansion is essential for unravelling invasion mechanisms and developing refined sustainable pest management strategies [5,6].

*Zeugodacus tau* (Walker), commonly referred to as the pumpkin fruit fly, is a highly mobile invasive tephritid with a remarkable capacity for cross-family host shifts. Note: This species was previously published under the synonym *Bactrocera tau* in older taxonomic and ecological literature, following genus-level reclassification it is now formally assigned to *Zeugodacus*. It is a highly mobile invasive tephritid with a remarkable capacity for cross-family host shifts [7]. Though evolutionarily specialized to feed on Cucurbitaceae crops, *Z. tau* has expanded its host spectrum to more than 80 plant taxa, including numerous commercially valuable fruit species from divergent plant families [8,9]. Climate warming fuels the global expansion of polyphagous tephritid pests, with China serving as a representative regional case for *Z. tau*. Ongoing climate warming accelerates its northward range expansion within this country, where field surveys have confirmed established populations in northern provinces such as Shanxi and Shaanxi [10,11]. Ecological modelling further predicts continuous spread into domestic temperate vegetable and fruit-growing zones, substantially raising invasion risks for local cultivated crops [12].

Host shifts by this pest trigger widespread agricultural economic losses in its distribution range; for example, domestic annual economic losses within China have been estimated at 3.7 × 10^7^ to 2.3 × 10^10^ yuan [13]. Current control practices predominantly depend on synthetic insecticides, mass trapping and cultural interventions [14,15]. Nevertheless, the broad polyphagy of *Z. tau* restricts targeted suppression measures, while overreliance on chemical pesticides accelerates pest resistance and compromises food safety and ecosystem integrity [16]. There is therefore an urgent demand for eco-friendly monitoring and control tools tailored to this polyphagous fruit fly [17].

As an obligate fruit-boring species, all larval life stages of *Z. tau* develop entirely within fruit pulp [18,19]. Consequently, larval tolerance to fruit chemical composition acts as the core determinant of successful host colonization. Unlike ancestral cucurbit hosts, the novel hosts this pest colonizes via host shift contain unique nutritional pools and defensive secondary metabolites that impose chemical stress on feeding larvae [20,21]. Host shift represents a bidirectional plant–insect interaction: fruit chemical signatures shape larval fitness performance, whereas larvae remodel endogenous metabolic networks to respond to unfamiliar host microenvironments [22]. Metabolic reprogramming serves as the primary adaptive machinery underlying the utilization of novel hosts, yet the metabolic crosstalk linking *Z. tau* larvae to alternative fruit hosts remains poorly resolved, hindering the design of precise species-specific control tactics [23].

Untargeted liquid chromatography–mass spectrometry (LC–MS) metabolomics enables holistic characterization of chemical interactions between herbivores and their host plants, capturing both nutrient assimilation and stress-induced metabolic remodelling [24]. Most existing omics investigations into tephritid host shifts rely on single-tissue unpaired profiling: studies either independently characterize fruit chemical profiles or endogenous physiological responses of flies [24,25,26,27,28,29], but rarely integrate matched metabolomic datasets from both host tissue and feeding larvae across divergent plant lineages. To date, host-specific diagnostic metabolites and core metabolic changes underlying host shifts in *Z. tau* remain uncharacterized via matched fruit–larva metabolomics.

Here, we aim to characterize host-correlated larval metabolic divergence after multi-generational laboratory acclimation on phylogenetically distinct fruits, and explore metabolic signatures linked to differential host suitability. To test this hypothesis, we established a matched fruit–larva untargeted LC–MS metabolomics workflow, combined with multi-generational host suitability fitness assays across eight host taxa, consisting of three ancestral cucurbit crops and five commercial fleshy fruits. We pursued four specific research objectives: (1) quantify larval fitness divergence across hosts after multi-generational acclimation; (2) screen differential metabolites and functional pathways correlated with long-term host rearing; (3) characterize two distinct metabolic signature profiles associated with ancestral versus novel fruit hosts, generalist metabolic strategy, or host-specialized metabolic strategy targeting a specific host; (4) provide correlative biochemical data to inform sustainable pest management research.

This study advances correlative insights into host range expansion in polyphagous invasive tephritids. By identifying host-specific metabolic putative biomarker candidates and divergent larval adaptive pathways, our matched dual-metabolome dataset delivers empirical evidence to inform field pest surveillance and semiochemical lure development, supporting low-input, diversified horticultural pest management.

## 2. Materials and Methods

### 2.1. Insects and Host Fruits

Wild-collected *Z. tau* were cultured for subsequent trials. Full rearing protocols and environmental parameters are provided in Supporting Information Methods S1. For this laboratory-rearing experiment, a single host-adapted colony was established for each host plant. All biological samples for fitness and metabolomic analyses were obtained from successive generations of this single lineage. Accordingly, the replicates in this study are within-lineage biological replicates and do not constitute independently founded random populations. We recognize that pseudoreplication cannot be avoided under this experimental design; this critical limitation is explicitly addressed in Section 4.

Ten fruit types were chosen based on published host records and preliminary olfactory bioassays [30,31,32]. The panel included three ancestral cucurbit hosts: pumpkin (*Cucurbita moschata*), summer squash (*Cucurbita pepo*), and cucumber (*Cucumis sativus* L.). The remaining seven fruits from phylogenetically divergent plant families consisted of four confirmed novel commercial hosts (banana *Musa paradisiaca*, mango *Mangifera indica* L., sweet orange *Citrus sinensis* Osbeck, guava *Psidium guajava*) plus three potential subtropical and temperate hosts (pitaya *Hylocereus undatus* (Haw.) Britt. and Rose, apple *Malus pumila* Mill., peach *Prunus persica*). Olfactory assays demonstrated attractiveness of the three potential fruits, which were therefore assessed for their capacity to support host shifting in this study.

All host fruit common-names and species identities have been standardized throughout the manuscript, figures and supplementary tables. For each fruit host, verified scientific names, commercial cultivars, fruit origin, ripeness judging criteria, storage duration, and the number of independent fruits used for assays are summarized in Supporting Information Table S1. Note that “orange” was incorrectly labelled as “Sweet orange” in Figure 2; this label has been corrected.

Apple and peach were included only for life-history fitness bioassays, whereas they were excluded from formal untargeted metabolomic data acquisition. Figure S6 displays preliminary exploratory metabolite profiling data from these two hosts, which were not incorporated into the main metabolomic statistical analysis.

All experimental fruits were fully naturally ripened with consistent texture and characteristic peel pigmentation. Fruits were stored at 4 °C (24–48 h) and acclimated to room temperature (25 ± 0.5 °C prior to experimental setup).

### 2.2. Host-Shift Assay

A host-shift experiment was conducted to evaluate the host range expansion potential of *Z. tau*. All host experimental lines were initiated in parallel from the identical pumpkin base population to avoid initial genetic bias. Each host species was maintained in three parallel rearing cages derived from the same host-specific founding lineage throughout multi-generational acclimation; no sequential cross-host transfers were performed during rearing, thereby preventing population bottlenecks.

Each experimental cage contained 40 mature adults (20 females and 20 males). Sliced fruits were supplied for adult feeding, and intact whole fruits were provided as oviposition substrates. Fly populations on each candidate host were continuously reared for at least three successive generations.

The metric termed host colonization establishment time is a population-level composite index, rather than a standard individual-level developmental or generation-time measurement. It quantifies the time interval from initial adult exposure to host fruit until the colony successfully produces more than 30 newly emerged adults. This metric integrates oviposition, egg hatching, larval-pupal development, adult emergence, and overall colony-establishment performance. The observed range of 112–145 days reflects the total time required for successful host-associated colony establishment across treatments and does not represent single-generation individual development. Initial adult numbers per replicate, fruit quantity, fruit-replacement frequency, and the origin of biological replicates are summarized in Supporting Information Table S2. Owing to experimental constraints, initial egg and larval densities could not be precisely standardized across host groups; this limitation is further addressed in Section 4.

Apple and peach enabled female oviposition but failed to sustain stable multi-generational colony establishment over repeated transfers. Accordingly, these two hosts were only used for life-history fitness assays and excluded from matched fruit–larva metabolomic profiling. Finally, eight host species were retained for downstream metabolomics: three ancestral cucurbit hosts and five non-cucurbit fleshy fruits (four verified novel commercial hosts plus pitaya as a candidate potential host).

### 2.3. Evaluation of Host Adaptability

Host adaptability was assessed based on colonization success, developmental duration and larval fitness. The time required for flies to complete the full life cycle (egg to adult) and produce more than 30 newly emerged adults on each host was defined as host colonization establishment time. We note that this defined “colonization establishment time” is a composite index rather than a standard metric of individual developmental rate. This parameter integrates multiple components including larval development speed, survival, reproductive output and colony establishment success.

All third-instar larvae used for fitness measurements originated from F3 to F5 host-acclimated lines. Importantly, larvae sampled for subsequent metabolomic profiling were sourced from the same F3–F5 host-adapted populations employed for fitness evaluation.

Fitness assays adopted three within-lineage biological replicates per host. Three fitness indices were quantified: individual pupal weight, total number of pupae, and pupal emergence rate. We note that colonization establishment time, pupal fresh weight and adult emergence rate mainly reflect larval post-hatching performance. The total number of pupae represents a composite outcome shaped jointly by adult oviposition preference, egg viability and larval survival; therefore, this metric cannot be interpreted solely as a direct measure of larval fitness, but may serve as an indirect composite parameter reflecting overall host suitability for *Z. tau*.

Larvae from F3–F5 generations of each host-reared colony were sampled for fitness assays and untargeted metabolomic profiling. Measurements collected across F3–F5 generations were averaged to comprehensively evaluate host adaptability.

All statistical analyses were performed using Origin 2024. Data are presented as mean ± standard error (SE). Independent *t*-tests compared host colonization establishment time of each alternative host against pumpkin (ancestral host). One-way ANOVA was used to detect inter-host differences in fitness indices. Significance thresholds were set as * *p* < 0.05, ** *p* < 0.01 and *** *p* < 0.001.

No formal multiple-testing correction was applied for pairwise comparisons across hosts and fitness traits; these comparisons are treated as exploratory rather than strict hypothesis testing. Several analyses are based on only three biological replicates, so inferences about host-suitability gradients are regarded as preliminary. For pupal-count data, interpretation is confounded by unstandardized natural oviposition pressure across hosts. Emergence rate was calculated as the proportion of successfully emerged pupae; binomial-model analysis was not implemented in this study.

### 2.4. Metabolomic Analysis

#### 2.4.1. Sample Preparation

Untargeted metabolomic profiling was conducted for eight host fruits and their corresponding *Z. tau* larvae, with four biological replicates per treatment. Fruit tissue samples weighed 200–500 mg depending on fruit moisture content. F5 third-instar larvae continuously reared on each host for five generations were harvested, with roughly 200 mg larval tissue per replicate. A total of 64 samples (32 fruit, 32 larval) were preserved at −80 ± 0.5 °C; full sample metadata are provided in Table S1. Each larval metabolomic replicate comprised 15 pooled whole third-instar larvae, which were size-sorted to standardize developmental status. Larvae received only brief surface rinsing; no starvation period, gut clearance, or tissue dissection was performed before metabolite extraction. Fruit and larval samples were batch-matched rather than collected from identical individual fruits; full sample-to-batch correspondence is provided in Table S2. Gut-contained food residues may shape larval metabolomic profiles, and this confounding factor is noted in Section 4. All third-instar larval samples collected for untargeted metabolomic profiling were mixed-sex individuals, and no sex screening was performed prior to metabolite extraction. During multi-generational rearing and sample collection, larval density inside each fruit was maintained at a uniform level across all eight host species to avoid metabolic shifts driven by variable crowding pressure.

For each host, fruit and larval samples were collected from parallel batches under identical rearing conditions, but individual larval replicates were not sampled from the exact same fruit individual used for fruit metabolome measurement. Each larval metabolomic replicate consisted of 15 pooled whole third-instar larvae; only actively feeding larvae at identical developmental status were selected. Larvae were briefly surface-rinsed with pre-cooled ultrapure water to remove surface fruit debris, but no starvation, gut clearance or dissection was performed prior to metabolite extraction. Consequently, ingested fruit materials within the gut may contribute to the observed larval metabolomic profiles. A complete sample-matching table with pairing identifiers for fruit and larval samples is provided in Table S2. Since larvae and fruit were from matched batches rather than one-to-one same-fruit sampling, conventional paired statistical tests were not applied.

#### 2.4.2. Metabolite Extraction and UPLC-MS Detection Conditions

Metabolites were extracted using a mixed solvent system consisting of methanol:acetonitrile:water (2:2:1, *v*/*v*/*v*) spiked with isotopically labelled internal standards for quantitative normalization. Briefly, subsamples of 20–50 mg fruit tissue and 25 mg larval tissue were taken from the frozen specimens described above, homogenized, vortexed, and ultrasonicated on ice to facilitate metabolite release. The extraction cycle was repeated three times to maximize metabolite recovery. All processed samples were incubated at −40 °C for 1 h, followed by centrifugation at 12,000 *g* for 15 min at 4 °C. The resulting supernatants were collected and retained for untargeted metabolomic profiling.

Metabolite detection was performed using a Vanquish UPLC system (Thermo Fisher Scientific Inc., Waltham, MA, USA) coupled to an Orbitrap Exploris 120 mass spectrometer(Thermo Fisher Scientific Inc., Waltham, MA, USA) (Thermo Fisher Scientific). Full chromatographic and mass spectrometric parameters are provided in Supporting Information Methods S2, and information for key metabolites is listed in Table S3.

#### 2.4.3. Metabolome Data Processing

Thirty-two fruit samples corresponding to three cucurbit and five non-cucurbit fleshy host species were analyzed. Group abbreviations are defined as follows: NG (pumpkin), XHL (summer squash), HG (cucumber), XJ (banana), MG (mango), JZ (sweet orange), FSL (guava), and HLG (pitaya). Raw UPLC-MS spectra were converted to mzXML format using ProteoWizard v3.0.7414. Subsequent data processing, metabolite annotation and statistical analyses were conducted in R (caret package v6.0.90) with metabolite matching against the BiotreeDB v3.0 database [33]. Full analytical pipelines and screening criteria are provided in Supporting Information Methods S3.

The 32 larval metabolomic samples were processed using identical chromatographic and bioinformatic workflows and assigned corresponding labels: ZNG, ZXHL, ZHG, ZXJ, ZMG, ZJZ, ZFSL, and ZHLG. Differential metabolites were screened by integrating variable importance in projection (VIP) from PLS-DA, fold change and one-way ANOVA *p*-values. DAMs were preliminarily screened using thresholds: VIP > 1, |fold change| > 2 and raw *p* < 0.05. Notably, no FDR correction for multiple testing was applied in this exploratory untargeted metabolomic survey. We acknowledge that metabolites with marginal statistical significance may represent false positives. Accordingly, mechanistic interpretation within the manuscript primarily focuses on core metabolites exhibiting substantial and consistent abundance changes well above the screening thresholds.

All retained DAMs were annotated and mapped to the KEGG database for functional classification and pathway enrichment. Combined with previously published metabolic evidence, these datasets were used to interpret key host–larva metabolic associations and candidate adaptive pathways linked to host range expansion in *Z. tau*.

Quality control (QC) samples were evenly injected throughout the analytical sequence. Highly overlapping total ion current (TIC) profiles and relative standard deviation (RSD) < 15% for metabolite peak intensities confirmed stable instrument performance and good reproducibility of metabolomic measurements. All QC samples were prepared by pooling equal volumes of extracts from all fruit and larval sample treatments; no additional mathematical drift correction algorithms were applied apart from the QC-based instrumental stability inspection.

Pairwise correlation analysis of key metabolites between host fruits and *Z. tau* larvae was conducted using Pearson correlation. Correlation coefficients and corresponding *p*-values were calculated based on four biological replicates per host species. Metabolite abundance values at the sample level were log-transformed before correlation analysis.

## 3. Results

### 3.1. Host Adaptability of Z. tau Across Distinct Host Plants

Several life-history parameters were measured to assess host-associated performance. It should be noted that total pupal number is a composite variable influenced by multiple life stages rather than a pure indicator of larval fitness.

Host suitability and host-shift capacity of candidate plant species were evaluated for *Z. tau*. Though apple and peach received ovipositional punctures from adult flies, these hosts failed to support complete larval development and therefore could not sustain stable field populations. Eight plant species were validated as viable developmental hosts: three cucurbit crops (pumpkin, summer squash, cucumber) and five commercial fleshy fruits, comprising four novel hosts (banana, mango, orange, guava) and one potential host (pitaya), all capable of sustaining continuous *Z. tau* colonies.

**Figure 1:**
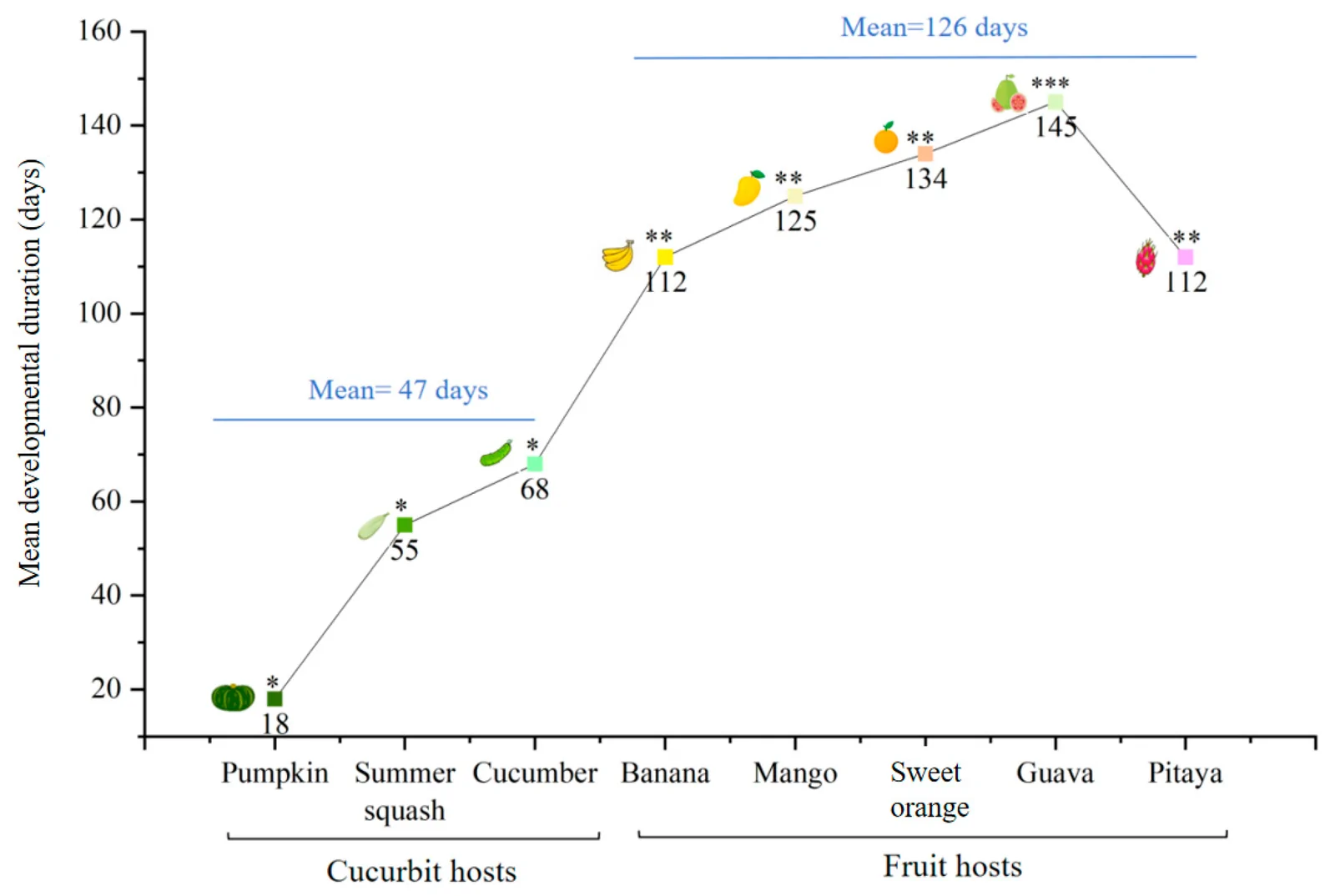
Mean colonization establishment time of *Z. tau* on different host plants. This metric quantifies the time interval from initial adult exposure to host fruit until the colony successfully produces more than 30 newly eclosed adults. Values represent means ± SE of three biological replicates. * *p* < 0.05, ** *p* < 0.01, *** *p* < 0.001, compared with the ancestral pumpkin host. One-way ANOVA: F = 8.72, df = 7,16, *p* = 0.0004.

Colonization establishment time of *Z. tau* differed markedly among plant groups (Figure 1). Populations established far more rapidly on cucurbit hosts, with a generational cycle averaging 47 days, versus 126 days for fruit hosts (*p* < 0.001). Pumpkin supported the shortest full generation time at 18 days. Relative to pumpkin, development was moderately extended on summer squash and cucumber (*p* < 0.05), with no statistical divergence detected between these two cucurbit species. Among fruit hosts, generational duration followed the ascending order: banana and pitaya (112 d), mango (125 d), orange (134 d), and guava (145 d) (Figure 1). All fruit hosts exhibited significantly prolonged developmental cycles compared with pumpkin (*p* < 0.01 for most fruits; guava, *p* < 0.001).

**Figure 2:**
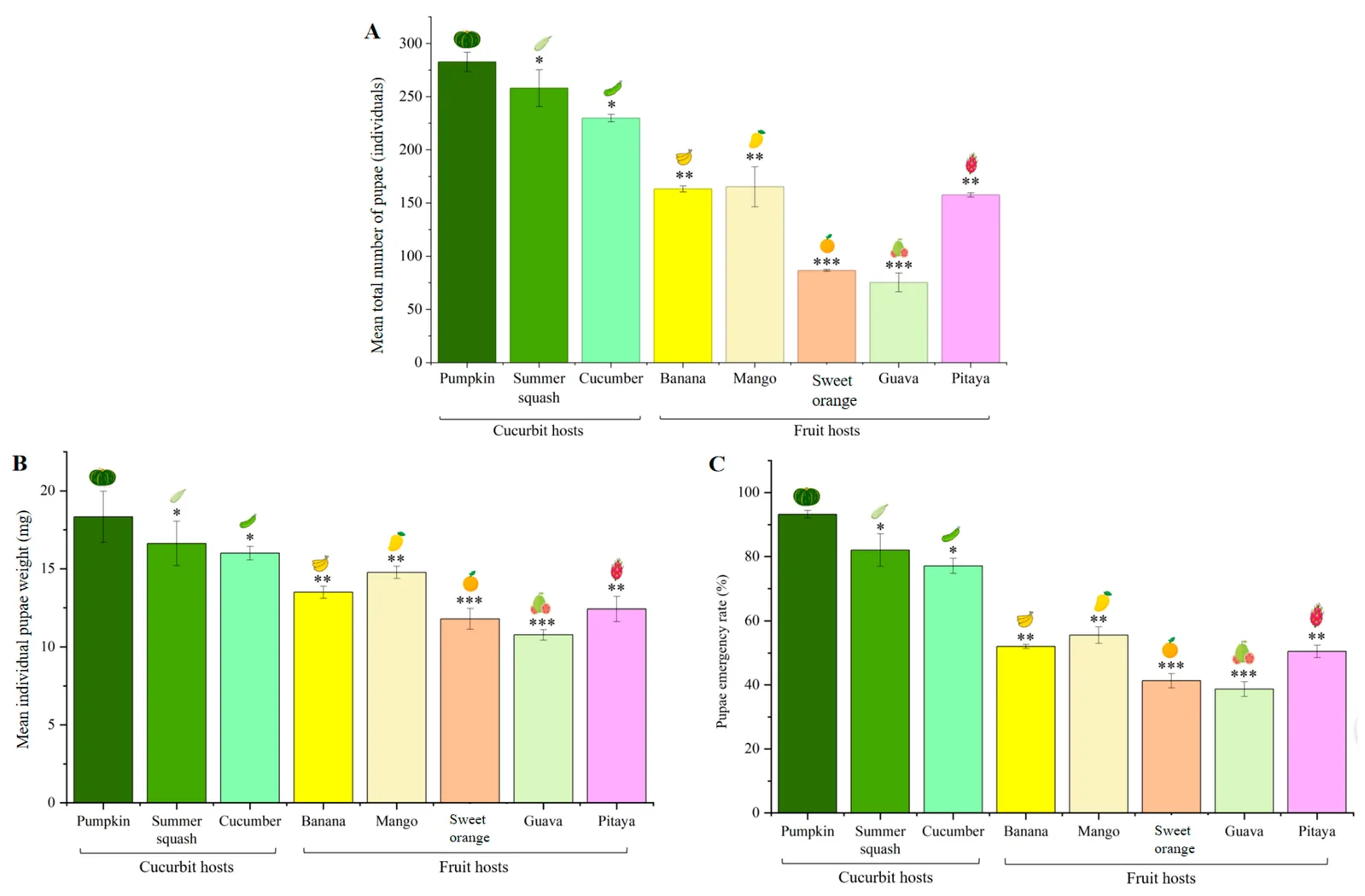
Life-history fitness traits of *Z. tau* fed on various hosts: (**A**) total number of pupae; (**B**) individual pupal weight; (**C**) pupal emergence rate. Data are means ± SE from three biological replicates. Significance levels marked relative to pumpkin host: * *p* < 0.05, ** *p* < 0.01, *** *p* < 0.001.

Three core larval fitness metrics were quantified relative to the ancestral pumpkin host control (Figure 2): mean individual pupal weight (PW), mean total number of pupae (PN), and pupal emergence rate (ER). Flies reared on pumpkin exhibited the maximum PW (17 mg), PN (278 individuals), and ER (94%). All alternative hosts corresponded to significantly reduced fitness performance in *Z. tau*. The remaining two cucurbit host species displayed moderate fitness reductions (*p* < 0.05); banana, mango and pitaya showed stronger inhibitory impacts (*p* < 0.01); orange and guava yielded the most pronounced fitness suppression (*p* < 0.001) (Figure 2).

Integrating colonization success, colonization establishment and larval-related fitness metrics, our results demonstrate that *Z. tau* exhibits the greatest adaptive performance on ancestral cucurbit hosts. Moderate host adaptation was observed on the novel fruit hosts banana and mango, as well as the potential host pitaya, whereas orange and guava represented relatively low-suitability hosts with relatively weak supporting capacity for this tephritid pest.

### 3.2. Host Fruit Metabolome

#### 3.2.1. Overview of Hosts Metabolite Profiles

**Figure 3:**
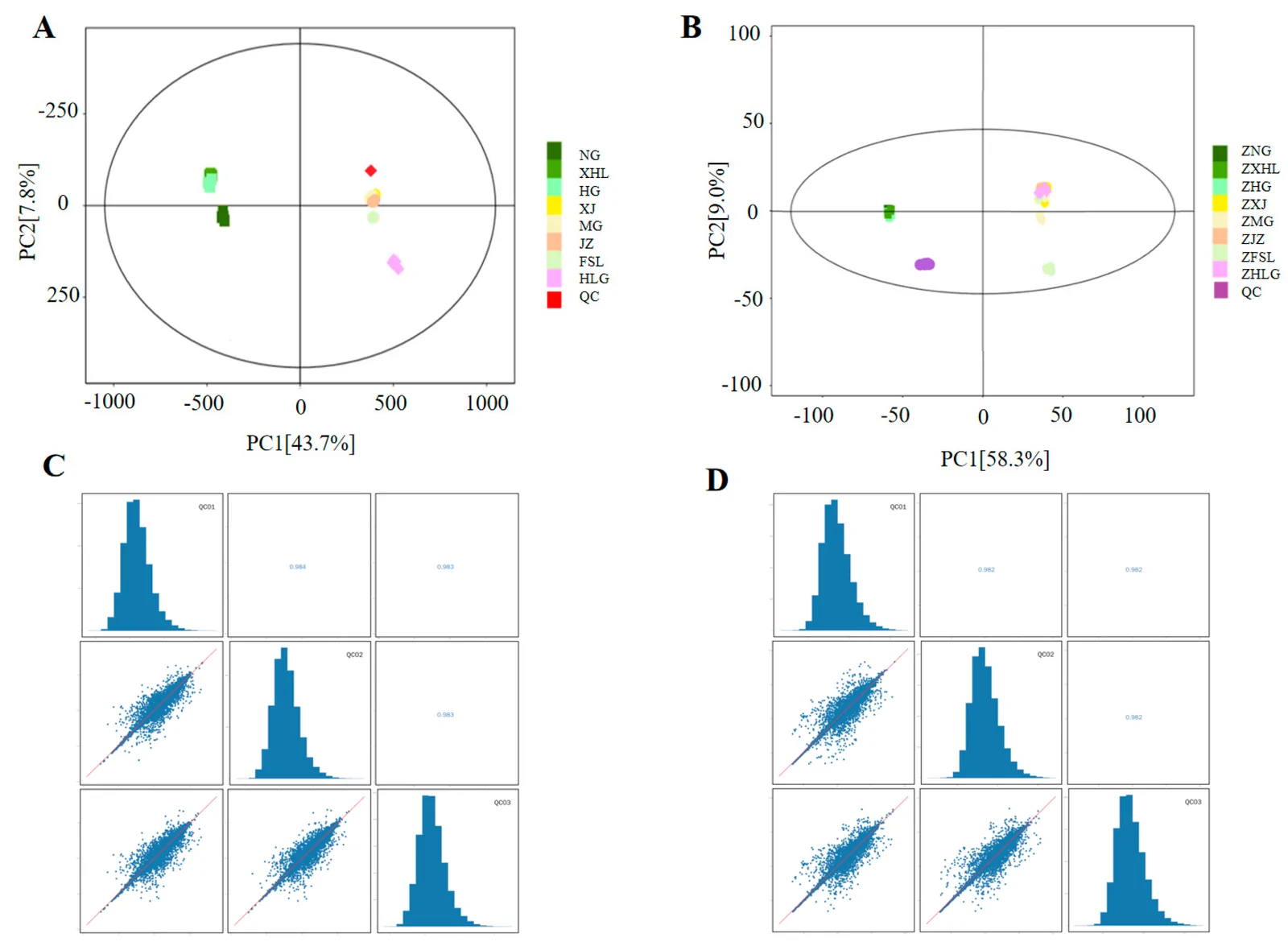
Principal component analysis and quality control assessment of fruit and larval metabolomic datasets. (**A**) PCA score plot showing global metabolic separation among eight host fruit groups. The 95% confidence ellipses are drawn to delineate the distribution range of each host group. PC1 and PC2 explain 43.7% and 7.8% of total variance, respectively. (**B**) PCA score plot for *Z. tau* larvae reared on different host fruits. The 95% confidence ellipses delineate the distribution range of each larval group. PC1 and PC2 explain 58.3% and 9.0% of total variance, respectively. Pooled-QC assessment panels display pairwise Pearson-correlation scatter plots and feature-intensity histograms. (**C**) For fruit-derived pooled-QC samples, pairwise Pearson correlation coefficients ranged from 0.983 to 0.984; (**D**) for larval pooled-QC samples, pairwise Pearson correlation coefficients were consistently 0.982. These values demonstrate high instrumental reproducibility of the LC-MS profiling.

A total of 2398 metabolites were annotated across the eight tested host fruits, which were classified into eight primary, 59 secondary and 246 metabolite classes (Figure S1A). Principal component analysis (PCA) was performed to visualize global metabolic separation among host groups, with biological replicates enclosed within 95% confidence ellipses for each group. The first two principal components (PC1 and PC2) accounted for 43.7% and 7.8% of the total variance, respectively (Figure 3A). All host samples were clearly divided into two distinct clusters along the PC1 axis: the three cucurbit hosts (NG, XHL, HG) with highly similar metabolic profiles (PC1 < 0), and the five fruit hosts (XJ, MG, JZ, FSL, HLG) with divergent metabolic features (PC1 > 0) (Figure 3A). Instrument reproducibility of the metabolomic profiling was supported by pooled-QC sample pairwise Pearson correlation coefficients ranging from 0.983 to 0.984 (Figure 3C), indicating stable LC-MS acquisition for fruit and larval datasets.

#### 3.2.2. Differential Metabolites

**Figure 4:**
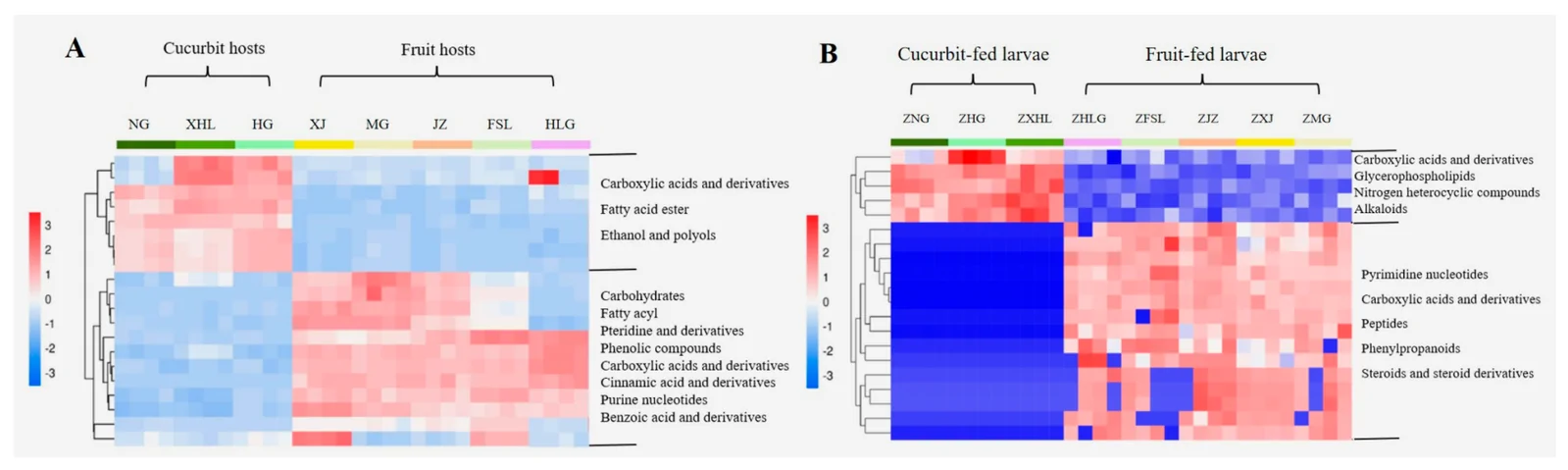
Hierarchical clustering heatmaps of differentially accumulated metabolites from host fruits and corresponding larvae. (**A**) Metabolite profiles of eight host fruits. (**B**) Profiles of *Z. tau* larvae reared on distinct hosts. Red denotes high relative metabolite abundance, while blue denotes low abundance. Host and larval abbreviations are defined in Materials and Methods. The colour scale displays standardized Z-values from −3 to 3. Consistent with PCA results, cucurbit and fleshy fruit hosts formed separate clusters in both fruit and larval metabolomic datasets.

**Figure 5:**
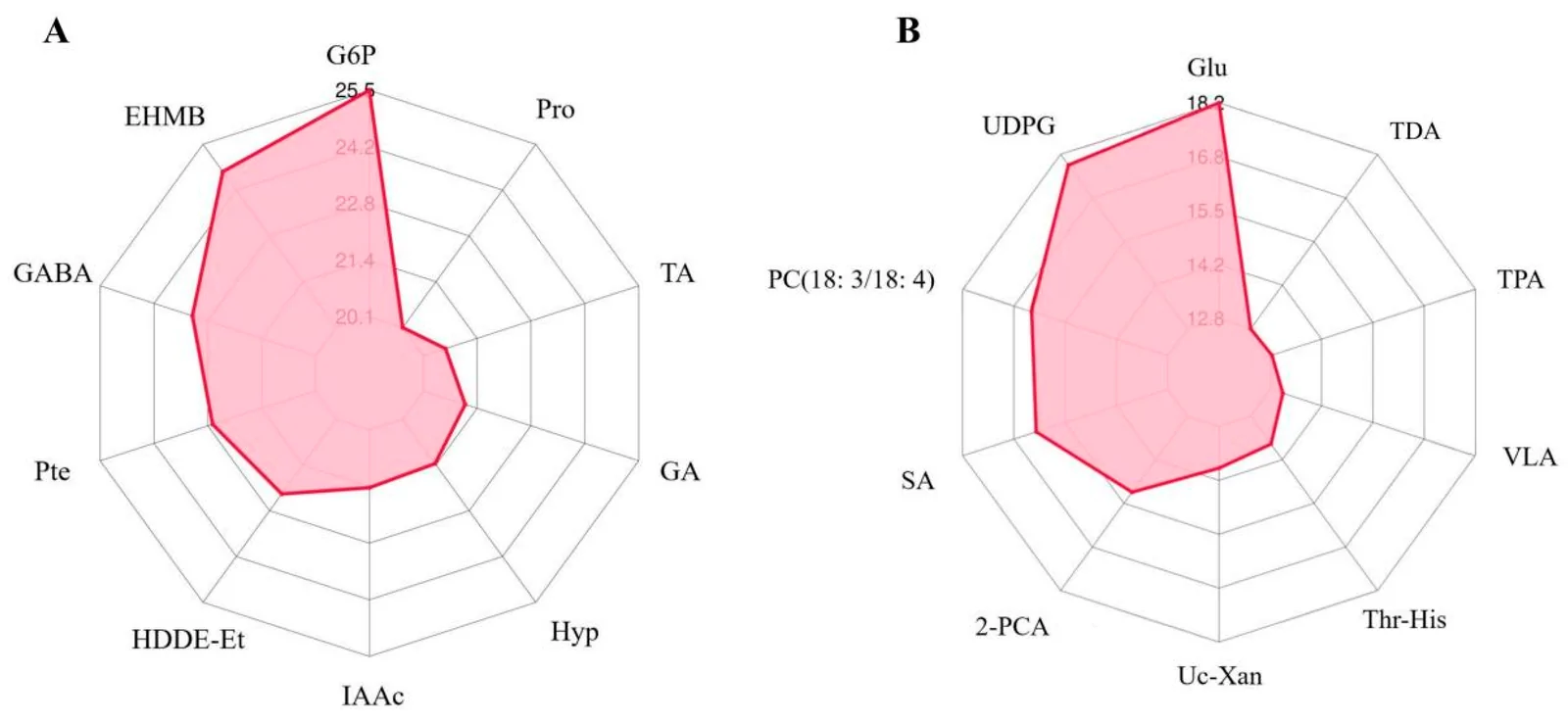
Radar charts for the ten most significantly differentially accumulated metabolites. Radar chart illustrating statistical significance of metabolite differences across host treatments (−log_10_ *p*). This plot depicts significance levels only and does not reflect metabolite abundance. Corresponding metabolite relative-abundance profiles are shown in Figure 6 and Figure 7. (**A**) Metabolic variation across eight host fruits. (**B**) Metabolic variation in *Z. tau* larvae. Each axis corresponds to one metabolite, scaled by the −log_10_ *p* of one-way ANOVA. Concentric circles mark graded values, with larger values farther from the centre denoting stronger statistical significance and greater metabolite divergence among host groups. Red shading highlights metabolites with significant differential accumulation. All metabolite abbreviations are listed in Table 1.

**Figure 6:**
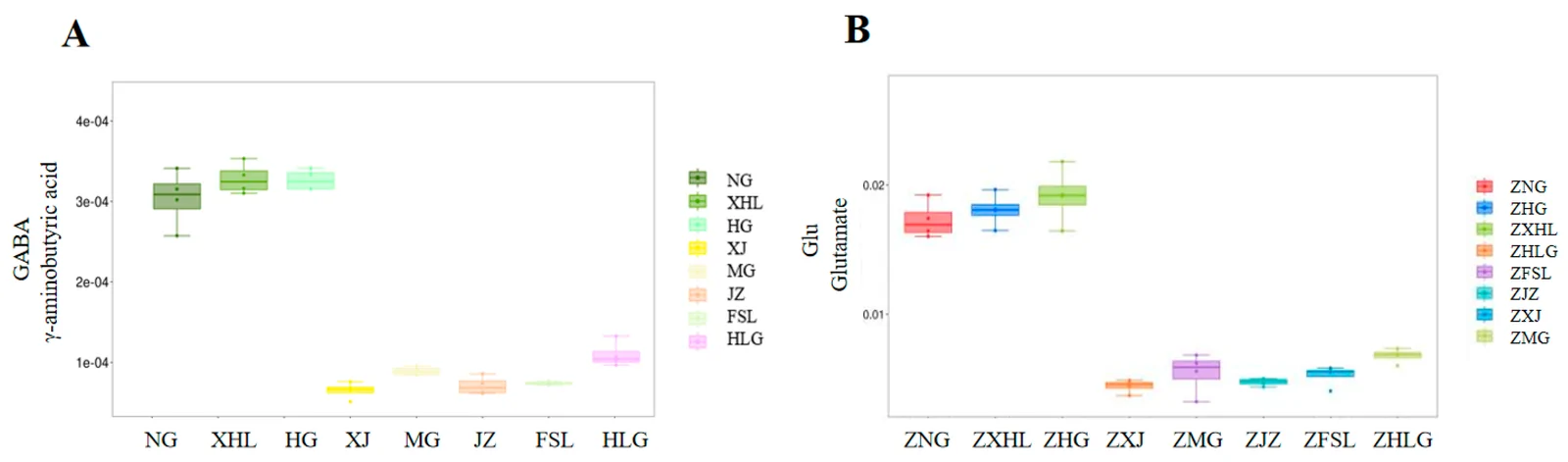
Box plots of relative abundances of key metabolites in cucurbit fruits and associated *Z. tau* larvae. (**A**) γ-aminobutyric acid (GABA) abundance across various cucurbit hosts; (**B**) glutamic acid (Glu) abundance in larvae reared on cucurbits. Boxes cover the 25th–75th percentiles, central lines denote medians, whiskers extend to min/max values, and scattered points stand for independent biological replicates.

**Figure 7:**
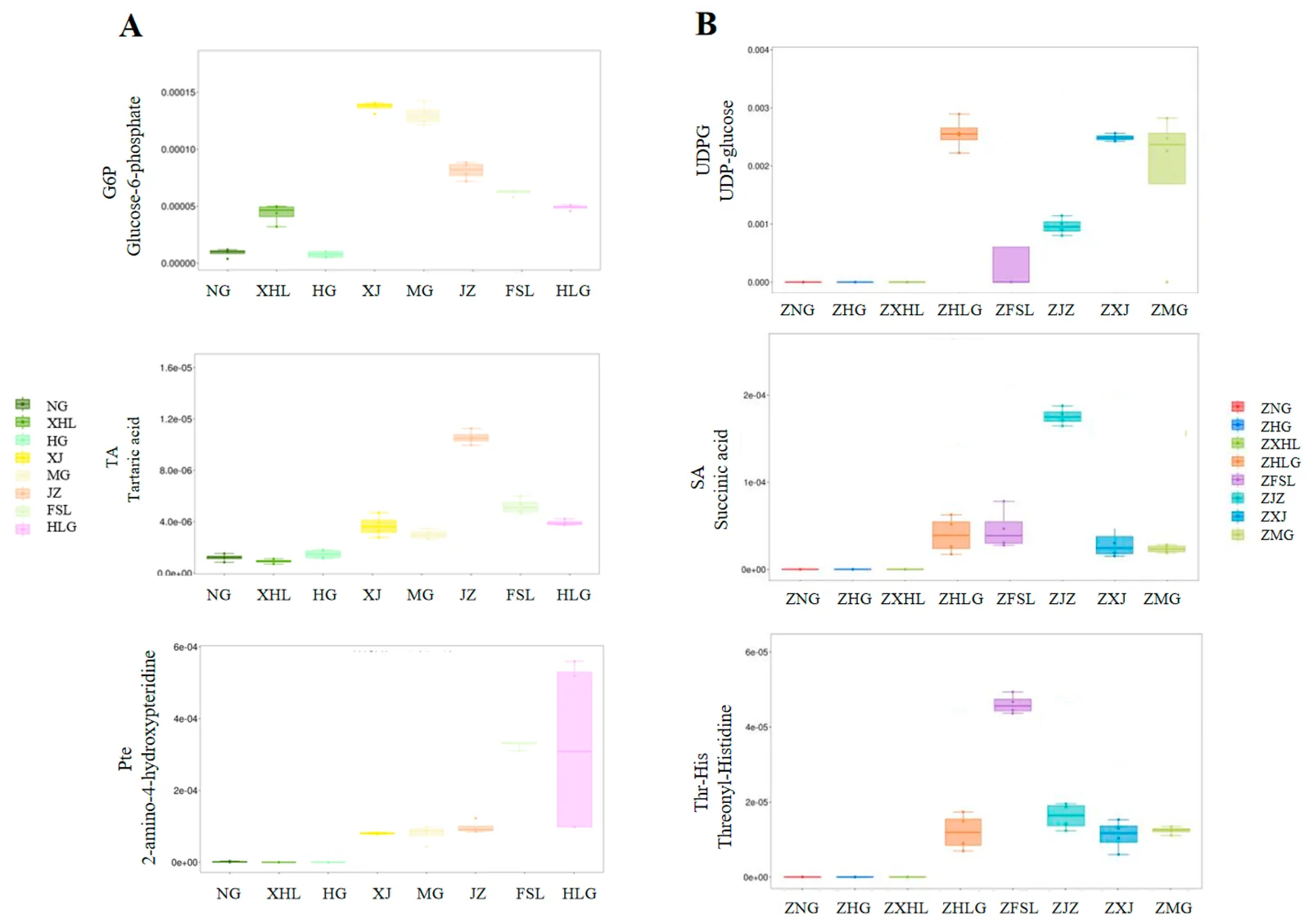
Box plots of key differentially accumulated metabolites in fleshy fruits and their corresponding *Z. tau* larvae. (**A**) Fruit metabolites: glucose-6-phosphate (G6P), tartaric acid (TA), 2-amino-4-hydroxypteridine (Pte). (**B**) Larval metabolites: UDP-glucose (UDPG), succinic acid (SA), threonyl-histidine (Thr-His). The y-axis shows relative metabolite abundance; individual dots represent biological replicates. Box plot styles match those used in Figure 6.

The heatmap of differential metabolites further validated the two-group separation revealed by PCA (Figure 4A). Obvious differences in metabolite enrichment were observed between cucurbit and fruit hosts. Cucurbit hosts were predominantly enriched in carboxylic acid derivatives, fatty acid esters, ethanol and polyols. By contrast, fruit hosts accumulated abundant carbohydrates, fatty acyl, pteridines, phenols, and nucleotide derivatives. A radar chart was used to visualize the statistical significance of metabolite differences across host treatments (plotted as −log_10_ *p*-values). Based on −log10 *p*-values ranging from 20.1 to 25.5, we screened ten highly significant differential metabolites (Table 1 and Table S3) and visualized their distribution via radar charts (Figure 5A). Note that this radar chart only illustrates the magnitude of statistical significance and does not represent metabolite abundance; metabolite relative-abundance patterns are presented separately in Figure 6 and Figure 7.

Two host-specific putative biomarker candidates were abundant in cucurbit fruits: γ-aminobutyric acid (GABA, Figure 6A, *p* = 1 × 10^−23^, less than 0.001) and ethyl (2E,4Z)-hexadecadienoate (HDDE-Et, Figure S2, *p* = 1.26 × 10^−22^, less than 0.001). GABA had the highest −log10 *p*-values = 22.9 (22.8–24.2) (Figure 5A) and modulates insect nitrogen metabolism through the GABA shunt pathway, while HDDE-Et participates in lipid β-oxidation. The contents of both metabolites were significantly higher in cucurbit hosts than in fruit hosts (GABA: *p* = 1 × 10^−23^; HDDE-Et: *p* = 1.26 × 10^−22^).

The other eight major differential metabolites were exclusively detected in larvae reared on fruit hosts (*p* < 0.001). Note: “exclusively detected” here denotes markedly higher abundance in fruit-host-reared larvae; these metabolites yield near-zero signals approaching or below instrumental detection limits in cucurbit-fed larvae and are not biologically absent. Glucose-6-phosphate (G6P), with a maximum −log_10_ *p*-value of 25.5 (Figure 5A), was defined as the core putative biomarker candidate for fruit hosts. The remaining fruit-specific metabolites, namely EHMB (Ethyl 2-hydroxy-3-methylbutanoate), 2-amino-4-hydroxypteridine (Pte), isoamyl acetate (IAAc), hyperoside (Hyp), gallic acid (GA), tartaric acid (TA) and L-pyrrolidine-2-carboxylic acid (Pro) (Table 1 and Table S3, Figure 5A, the *p*-values ranged from 2.94 × 10^−26^ to 7.94 × 10^−21^, all less than 0.001), are functionally associated with glycolysis, folate synthesis, olfactory perception, antioxidant defence and amino acid metabolism.

Each fruit host possessed unique metabolic characteristics. Banana showed high abundance of G6P and IAAc; mango was enriched in G6P and Hyp; orange contained the highest TA concentration; guava accumulated considerable GA and Pte (Figure 7A and Figure S2); pitaya had high levels of Pte and Pro. In particular, EHMB was universally enriched across all five fruit hosts (Figure S2).

### 3.3. Larval Metabolome

#### 3.3.1. Overview of Larval Metabolite Profiles

A total of 2425 metabolites were annotated across all *Z. tau* larval specimens, which were classified into 16 primary, 160 secondary, and 377 tertiary metabolic categories (Figure S1B). Principal component analysis (PCA) was performed to visualize global metabolic separation among host-reared larval groups, with 95% confidence ellipses drawn to delineate the distribution range for each larval group (Figure 3B). PC1 alone accounted for 58.3% of total variance and clearly separated larvae originating from cucurbit hosts from those reared on commercial fruits, while PC2 accounted for 9.0% of total variance. Larvae feeding on cucurbits clustered on the negative PC1 axis (PC1 < 0), whereas fruit-feeding individuals distributed along the positive PC1 axis (PC1 > 0). Instrument reproducibility for the larval metabolomic dataset was supported by pairwise Pearson correlation coefficients of 0.982 among pooled-QC samples (Figure 3D). The hierarchical clustering heatmap generated from all 32 larval samples further recapitulated this distinct bipartite grouping pattern (Figure S3). Overall, the larval metabolic grouping was highly congruent with the separation signature observed in their respective host fruit metabolomes.

#### 3.3.2. Differential Metabolites

The heatmap of differential metabolites further validated the two-group clustering of larval metabolic profiles (Figure 4B). Metabolite enrichment patterns differed distinctly between groups: larvae from cucurbit hosts were predominantly enriched in carboxylic acids, glycerophospholipids, nitrogen-containing heterocycles and alkaloids, whereas fruit-feeding larvae accumulated large amounts of pyrimidine nucleotides, peptides, phenylpropanoids and steroids. We screened ten highly significant differential metabolites (the *p*-values ranged from 6.31 × 10^−19^ to 1.58 × 10^−13^, both less than 0.001) according to −log_10_ *p*-values (12.8–18.2) (Figure 5B).

Four signature metabolites were specifically enriched in larvae adapted to cucurbit hosts: glutamic acid (Glu), PC(18:3(9Z,12Z,15Z)/18:4(6Z,9Z,12Z,15Z)) (PC(18:3/18:4)), piperazine-2-carboxamide (2-PCA) and uncyclized xanthommatin (Uc-Xan) (Figure 5B, Table 1). Glu exhibited the highest −log_10_ *p*-value (18.2), representing the primary putative biomarker candidates associated with signatures of central nitrogen metabolism (Figure 5B). PC(18:3/18:4) contributes to lipid metabolism and membrane remodelling. 2-PCA participates in xenobiotic metabolism, and Uc-Xan is an essential intermediate in tryptophan metabolism. Box plots demonstrated that Glu abundance was significantly higher in cucurbit-reared larvae (*p* < 0.001) (Figure 6B).

The remaining six metabolites were characteristic of fruit-feeding larvae: UDP-glucose (UDPG), succinic acid (SA), threonyl-histidine (Thr-His), terephthalic acid (TPA), vanillyl lactic acid (VLA), and Torvoside_A (TDA) (Table 1). UDPG showed the highest statistical significance and acts as a critical glycosyl donor for carbohydrate metabolism. SA is a key metabolite in TCA cycle-mediated energy metabolism; Thr-His is associated with protein hydrolysis; VLA is derived from tyrosine metabolism; TPA and TDA function in xenobiotic detoxification. In particular, UDPG was highly abundant in larvae reared on banana, mango and pitaya; SA peaked in orange-reared larvae; and Thr-His was uniquely accumulated in guava-reared larvae (all *p* < 0.001) (Figure 7B).

#### 3.3.3. Larval Metabolic Pathway Enrichment

**Figure 8:**
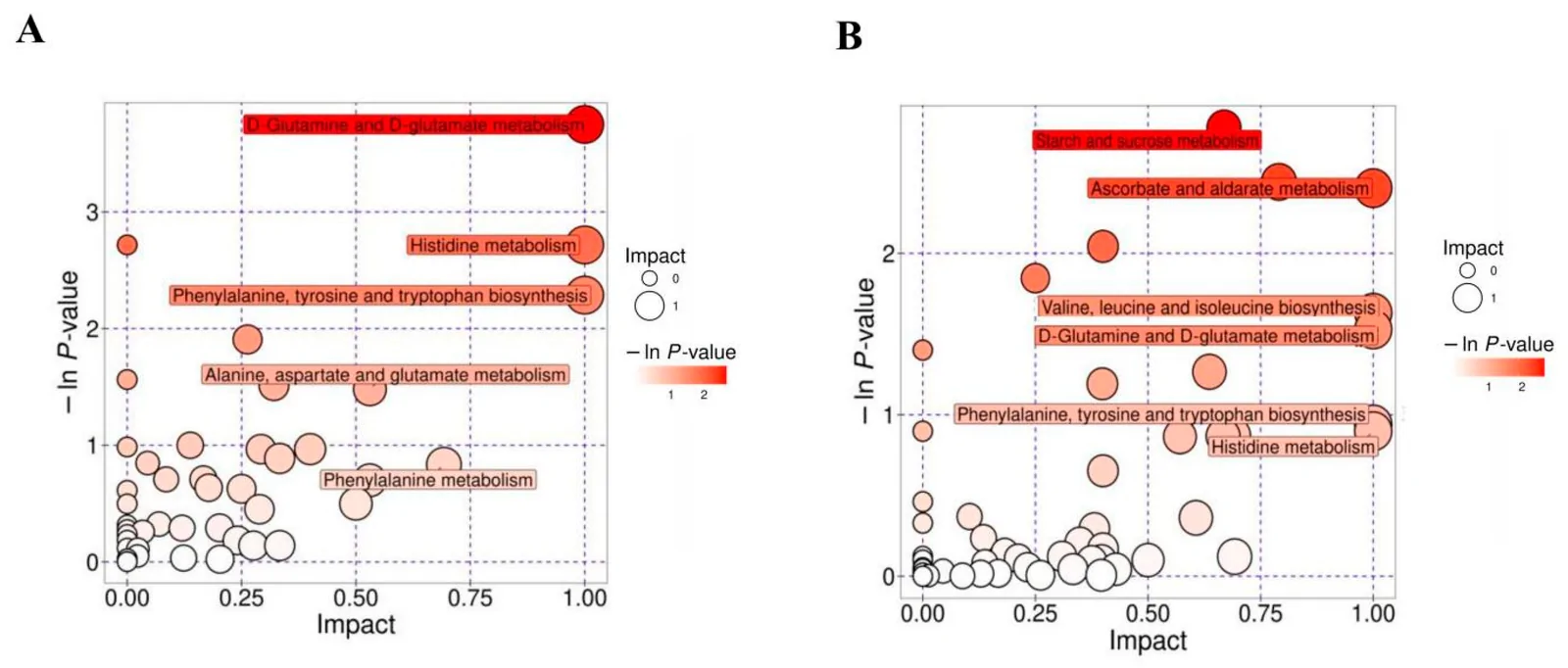
Bubble plots showing enriched metabolic pathways in *Z. tau* larvae. (**A**) Larvae feeding on cucurbit hosts. (**B**) Larvae feeding on fleshy fruit hosts. The x-axis shows pathway impact, representing topological weight within the metabolic network (larger values indicate greater pathway contribution). The y-axis displays enrichment significance, with higher values corresponding to smaller *p*-values. Bubble size equals the count of differential metabolites in each pathway. Bubble colour gradients from white to red denote increasing statistical enrichment significance.

KEGG functional annotation identified 14 significantly enriched metabolic pathways across the 32 larval samples (Figure S4), grouped into seven major functional categories: amino acid metabolism, carbohydrate metabolism, cofactor and vitamin metabolism, nucleotide metabolism, signal transduction, lipid metabolism, and transmembrane transport. In larvae fed cucurbit hosts, D-glutamine and D-glutamate metabolism displayed the strongest enrichment, followed by histidine metabolism, aromatic amino acid biosynthesis, and alanine–aspartate–glutamate metabolism (Figure 8A). By contrast, starch and sucrose metabolism represented the top enriched pathway in fruit-host larvae, alongside ascorbate and aldarate metabolism (Figure 8B). Distinct host-specific pathway signatures were detected within fruit-fed larvae: larvae reared on banana and pitaya exhibited dominant starch and sucrose metabolism (Figure S5A,B); larvae reared on mango showed strong enrichment of metabolites in ascorbate and aldarate metabolism (Figure S5C); orange exhibited prominent signatures in glyoxylate and dicarboxylate metabolism (Figure S5D); and guava-fed larvae showed the greatest enrichment of histidine metabolism (Figure S5E).

### 3.4. Associations Between Fruit and Larval Metabolism

Following multi-generational acclimation across eight host plant species, larval metabolomic clustering of *Z. tau* closely matched that of their corresponding host fruits. Both fruit and larval metabolic profiles segregated into two distinct clusters corresponding to cucurbit and non-cucurbit fruit hosts (Figure 3 and Figure 4), demonstrating coordinated host-specific metabolic divergence between the fly and its food source. The three cucurbit hosts exhibited highly similar metabolic signatures, with differential metabolites dominated by carboxylic acid derivatives, fatty acid esters and polyols. Two conserved diagnostic putative biomarker candidates, GABA (Figure 6A) and HDDE-Et (Figure S2), accumulated universally in all cucurbit fruits, enabling clear discrimination of cucurbits from other fruit types.

In response to the distinct chemical signatures of cucurbit fruits, *Z. tau* larvae exhibit striking accumulation of glutamate (Glu) in their metabolomes. The D-glutamine and D-glutamate metabolism pathway was broadly enriched among larvae reared on cucurbits (Figure 8A), and Glu stood out as the most significantly differentiated metabolite in larval samples (*p* < 0.001, −log_10_ *p* = 18.2; Figure 5B and Figure 6B). At the individual host species level, Pearson correlation analysis between fruit-derived GABA and larval Glu yielded a correlation coefficient of 0.52 and a *p*-value of 0.0001, with four biological replicates for each host species.

Drawing on locust metabolic models, GABA is hypothesized to reversibly convert to larval Glu via the GABA shunt, yet direct evidence for this reaction is absent in *Z. tau*. Additional amino acid-related pathways were also significantly enriched in cucurbit-fed larvae (Figure 8A). Collectively, these observations indicate that Glu-centred amino acid pathways are markedly enriched in cucurbit-feeding larvae; this correlative pattern reflects metabolic differences linked to host chemistry; functional experiments will be required to verify whether these pathway shifts contribute to host suitability. In contrast, non-cucurbit fruit hosts possessed highly heterogeneous metabolomes rich in carbohydrates, lipids, phenolic compounds and nucleotide derivatives, reflecting substantial interspecific variation in fruit chemical composition. Larvae feeding on these fruits exhibited a convergent metabolic pattern centred on starch and sucrose metabolism (Figure 8B). UDP-glucose (UDPG) served as a core metabolite shared by all fruit-adapted larvae (*p* < 0.001) and accumulated to high abundance in individuals reared on banana, mango and pitaya (Figure 5B and Figure 7B). Significant correlation was detected between larval UDPG and host glucose-6-phosphate (G6P; a metabolite biochemically related to UDPG), with a correlation coefficient of 0.68 and a *p*-value of 0.00001. This correlative relationship is consistent with sugar metabolism representing a primary host-associated pathway in *Z. tau* colonization across diverse fruit hosts.

While the core sugar metabolic pathway was conserved across all fruit-feeding larvae (Figure 8B), each host group displayed unique secondary metabolic features. Banana- and pitaya-reared larvae exhibited intensified starch and sucrose turnover (Figure S5A,B), whereas larvae reared on mango showed strong statistical enrichment within the ascorbate and aldarate metabolic module (Figure S5C). Elevated UDPG pools were observed in all three groups, consistent with active sugar metabolism. Banana and mango fruits accumulate abundant G6P (*p* < 0.001), which may be associated with larval carbohydrate metabolism (Figure 7A); additionally, mango-derived hyperoside (Hyp) may further regulate UDPG-associated metabolic networks.

The glyoxylate and dicarboxylate pathway, a vital gluconeogenic route representing an important component of carbohydrate metabolism, was uniquely enriched in orange-reared larvae (Figure S5D), with succinic acid (SA) as its pivotal metabolite (*p* < 0.001). Orange fruits contained high tartaric acid (TA) concentrations (Figure 7), which may represent a potential substrate source associated with larval glyoxylate cycle and downstream gluconeogenesis.

Larvae reared on guava exhibited specific enrichment of histidine metabolism linked to gluconeogenic processes (Figure S5E). Thr-His accumulated at significantly high levels in guava-reared larvae (*p* < 0.001), which may be associated with enhanced histidine catabolism. Guava fruits contained abundant Pte (Figure 7A,B), a metabolite that could modulate histidine degradation to support carbon skeleton production for gluconeogenesis.

The metabolic patterns observed in orange- and guava-reared larvae are consistent with increased reliance on gluconeogenic pathways, which may contribute to the reduced developmental fitness of these two low-suitability hosts. Fruits of guava accumulate high levels of Pte; this metabolite is tentatively hypothesized to modulate larval histidine metabolism linked to gluconeogenesis, though direct supporting evidence remains absent.

Collectively, *Z. tau* exhibits two divergent host-associated metabolic patterns across distinct host types. On chemically homogeneous cucurbit hosts, the fly shows enrichment of coupled Glu-centred amino acid pathways associated with host chemistry. Fleshy fruit-fed larvae display divergent carbohydrate metabolic signatures associated with host chemical composition after multi-generational laboratory rearing, covering starch–sucrose turnover, ascorbate and aldarate metabolism, and distinct gluconeogenic sub-pathways, which correlate with larval developmental performance across host types.

## 4. Discussion

Host shift represents a core adaptive strategy enabling invasive tephritids to expand their host ranges and exacerbate agricultural losses [34]. Unravelling the underlying physiological mechanisms is critical for designing targeted pest management tactics [16]. As an obligate fruit-boring insect, all larval developmental stages of *Z. tau* take place inside fruit tissues; consequently, larval tolerance to host chemical profiles largely influences successful colonization [20,21]. In the present work, untargeted LC–MS metabolomics was applied to simultaneously profile metabolites from eight host fruits and their corresponding larvae. Unlike prior tephritid metabolomic investigations that only characterized larvae alone [29,35], our matched dual-metabolome dataset synchronously links intrinsic fruit metabolic signatures to larval response pathways across eight phylogenetically distinct host species, offering a holistic view of host–larval metabolic crosstalk during host range expansion.

This untargeted matched fruit–larva metabolomics study reveals only correlative associations between host fruit chemistry and larval metabolic reprogramming. No functional manipulations, such as isotope tracing, gene RNA interference or enzyme activity measurements, were conducted in the current research. Accordingly, all inferred metabolic regulatory relationships cannot be confirmed as causal. Follow-up biochemical and genetic experiments will be necessary to distinguish passive nutrient uptake from active adaptive remodelling and to verify the biological function of candidate metabolic pathways.

This study is limited by the single-lineage rearing design for each host plant. All replicates were sampled from successively maintained laboratory colonies and thus represent intra-lineage variation rather than independent population replicates. Consequently, the observed metabolic and fitness differences may incorporate founder effects, genetic drift, and laboratory acclimation signals, and cannot be strictly generalized as general host-driven adaptation patterns.

Our population-level metric, host colonization establishment time, integrates multiple biological processes including oviposition success, egg survival and population proliferation, and cannot be interpreted solely as individual developmental rate. Unstandardized natural oviposition pressure across different hosts may confound the interpretation of pupal-count results, given that starting egg density was not manually controlled in this assay.

Caution should be exercised when interpreting similarities between fruit and larval metabolomes. Larval samples were whole-body extracts without gut removal, so detected metabolites may partly originate from ingested host-fruit material inside the gut, rather than purely endogenous larval metabolism. Accordingly, observed fruit–larva metabolic overlaps represent correlative patterns and cannot be directly interpreted as endogenous metabolic conversion without further functional validation.

Consistent with the phylogenetic constraint hypothesis [34,36], *Z. tau* established populations more rapidly and displayed higher fitness on ancestral Cucurbitaceae hosts relative to five commercial fleshy fruits. Apple and peach permitted adult oviposition but failed to support continuous larval growth and sustainable colony persistence. Metabolomic profiling uncovered marked chemical divergence between cucurbit and fleshy fruit hosts: cucurbit fruits accumulated abundant γ-aminobutyric acid (GABA) and lipid metabolites, whereas non-cucurbit fruits were rich in glucose-6-phosphate (G6P), flavonoids and organic acids. High levels of phenolic defensive compounds (Figure S6) in apple and peach likely suppress larval development [37], which may partially explain why *Z. tau* cannot complete its full life cycle on these two plant species. Collectively, interspecific metabolic differences among candidate hosts impose strong chemical selective pressure and fundamentally shape the host-shift potential and host breadth of *Z. tau*. These combined phenotypic and metabolomic observations support our central hypothesis that *Z. tau* larvae deploy flexible metabolic response strategies matching the distinct chemical microenvironments of novel host fruits to facilitate successful inter-family host colonization.

This study reveals marked divergence in host-associated metabolic profiles of *Z. tau* larvae responding to dissimilar fruit chemical environments. Larval metabolomic clustering closely aligns with host grouping, implying correlations between fruit chemical features and broad shifts in larval metabolic physiology. When reared on cucurbit crops, larvae exhibited elevated abundances of metabolites within D-glutamine and D-glutamate pathways, with glutamate (Glu) standing out as the most distinct differential metabolite. This larval glutamate enrichment coincides with high GABA abundance measured in cucurbit fruit tissues. Studies in locusts have demonstrated the presence of functional GABA-transaminase, the core enzyme of the GABA shunt pathway [38]. This pathway biochemically permits inter-conversion between GABA and glutamate, though we lack direct experimental evidence that dietary fruit-derived GABA undergoes such transformation in *Z. tau* larvae. In total, the Glu-centred amino acid metabolic pathway may be associated with the robust developmental performance of larvae reared on ancestral cucurbit hosts. Concurrent enrichment of glycerophospholipid and tryptophan metabolism could further sustain larval physiological homeostasis during feeding on cucurbit hosts.

For larvae developing on non-cucurbit fruits, starch and sucrose metabolism emerged as the primary host-associated metabolic module, with UDP-glucose (UDPG) identified as a key discriminatory metabolite. A strong positive correlation was observed between fruit glucose-6-phosphate (G6P) concentrations (banana, mango) and larval UDP-glucose (UDPG) levels (banana, mango and pitaya). This correlation is consistent with a potential association between fruit G6P availability and larval UDPG metabolism, but does not demonstrate uptake, transfer, or biosynthesis of fruit-derived G6P; the biochemical relatedness of G6P and UDPG is well established in insect carbohydrate metabolism [39,40,41]. This sugar-associated metabolic pattern may reflect larval acquisition of carbon nutrients and a potential response to chemical stress imposed by non-native fruit hosts. While core sugar metabolic frameworks are broadly conserved among fruit-feeding tephritid larvae, substrate-specific fine adjustments to metabolic profiles were detected across distinct fruit groups. Larvae exposed to oranges displayed elevated metabolites linked to the glyoxylate and dicarboxylate pathway—a canonical gluconeogenic branch representing an important component of insect carbohydrate metabolism [42]—within which succinic acid (SA) stood out as a prominent differential metabolite. High tartaric acid (TA) levels detected in orange tissues may induce shifts toward this alternative gluconeogenic route, possibly supporting basal metabolic turnover under nutritionally unfavourable conditions [42,43,44].

By comparison, larvae reared on guava exhibited enrichment of histidine metabolism linked to gluconeogenic processes [10], alongside elevated threonyl-histidine (Thr-His) that may facilitate histidine breakdown. High 2-amino-4-hydroxypteridine (Pte) abundance in guava fruit could further interfere with or reshape this histidine-centred metabolic network [45]. Larval metabolic profiles from orange and guava align with enhanced gluconeogenic pathway utilization, which may be associated with their weakened fitness performance. Guava-specific Pte may potentially alter larval histidine catabolism associated with gluconeogenic flux; this correlation remains speculative and requires dedicated feeding trials for verification. Elevated metabolite signatures of these gluconeogenic modules coincide with prolonged developmental duration, lower pupal mass and reduced emergence rates, consistent with increased reliance on energy-consuming gluconeogenic metabolism on suboptimal fruit hosts.

Notably, we detected no significant enrichment of classic cytochrome P450 or glutathione-dependent detoxification pathways in *Z. tau* at the metabolite level. This pattern differs sharply from most polyphagous folivorous herbivores, which rely heavily on these detox pathways to counteract plant defensive phytochemicals [26,46,47]. Such interspecies divergence may be linked to the species’ preference for ripe fruit tissues, which accumulate markedly lower levels of defensive secondary metabolites compared with vegetative plant parts [20]. It is plausible that constraints related to nutrient uptake and energy partitioning, rather than xenobiotic detoxification, may represent primary selective pressures during host colonization. However, this untargeted metabolomic dataset cannot rule out transcriptional or enzyme-level upregulation of detox machinery; P450 and GST activity may be modulated without producing detectable shifts in steady-state metabolite pools captured here. All detected differential metabolic pathways, whether derived from amino acid or carbohydrate metabolism, converge around the TCA cycle to support energy turnover, which suggests that balanced energy metabolism may be linked to larval developmental outcomes on novel suboptimal fruit hosts [27,48].

To our knowledge, few published studies have systematically performed matched metabolomic profiling of host fruits and *Z. tau* larvae across a gradient of host suitability. This work identifies two divergent response pathways together with core metabolic signatures linked to host acclimation, providing a foundational correlative framework for future functional characterization. Untargeted metabolomics offers a holistic overview of fruit–larva metabolic crosstalk and generates robust correlative evidence that helps to understand larval host-associated metabolic plasticity. Future efforts could explore the dynamic biochemical processes of host adaptation to disentangle causal regulatory relationships behind coordinated metabolic shifts between hosts and larvae. Detailed descriptions of analytical methods and biochemical validation in the Supporting Information (such as isotopic tracing validation) will help consolidate the inferred metabolic interconversion routes and enable standardized cross-study comparisons [49]. Collectively, this multi-host metabolomic dataset defines conserved core adaptive patterns and supplies promising candidate metabolic pathways to advance future research into host-shift mechanisms.

The putative biomarker candidates and host-associated metabolic pathways identified in this study offer potential biochemical insights for future development of low-input, precision pest management strategies targeting *Zeugodacus tau*. We outline two core translational directions as testable hypotheses generated from our metabolomic observations; additional extended prospects are framed as unvalidated hypotheses requiring dedicated follow-up experimental validation:

(1) Early pest risk forecasting: Host-associated metabolic markers (GABA in cucurbits, G6P in fleshy fruits) represent candidate indicators for future evaluation of host-colonization risk. A high fruit G6P/GABA ratio may hypothetically indicate elevated colonization risk for fleshy fruit hosts and could potentially assist orchard monitoring systems. This tentative inference arises from distinct metabolic signatures observed across the eight host species tested in our laboratory rearing system.
(2) Optimized attract-and-kill formulations: The observed correlation between fruit G6P and larval UDP-glucose is consistent with the hypothesis that G6P-related compounds might act as candidate phagostimulants to improve bait performance for fleshy fruit hosts; by contrast, glutamate (Glu) analogues could hypothetically disturb nitrogen metabolism in cucurbit-feeding larvae. These conceptual strategies are proposed based on conserved sugar and amino acid-related patterns observed from batch-matched fruit–larva metabolomes, and remain to be verified via behavioural bioassays and field trials.

The following ideas remain speculative hypotheses that could only be confirmed via dedicated laboratory and field trials, as relevant traits and practical effects were not measured within the present study:

(1) Exploration of plant-derived behavioural disruptors: Apple and peach contain phenolic compounds that suppressed larval development in fitness assays; these natural phenolics might act as eco-friendly antifeedants or repellents. Further lab bioassays and orchard tests are needed to verify their repellent activity.
(2) Implications for landscape configuration: Low-suitability hosts such as orange and guava are associated with enhanced gluconeogenesis and metabolic stress in larvae. Intercropping with these fruit types to constrain pest population expansion is merely a tentative concept and cannot be recommended for field application without systematic multi-season verification.

These translational prospects are derived solely from correlative metabolomic data; dedicated laboratory bioassays and multi-season field trials are essential to validate whether these metabolites can practically improve pest monitoring or suppression.

In summary, this metabolomic dataset delivers correlational evidence supporting biomarker-oriented strategies for sustainable *Zeugodacus tau* management. Further field trials investigating metabolite dose–response relationships could be conducted to translate laboratory observations into practical crop protection decision-making tools.

## Figures and Tables

**Table 1:** Detailed information of 10 differentially accumulated metabolites in hosts and larvae respectively.

Host Group	Name	Abbreviation	Subclass	Tertiary Class	Larvae Group	Name	Abbreviation	Subclass	Tertiary Class
Cucurbit Hosts	γ-aminobutyric acid	GABA	Carboxylic acid derivatives	Non-protein amino acids	Cucurbit Larvae	Glutamate	Glu	Carboxylic acid derivatives	Amino acids, peptides and analogues
						PC(18:3(9Z,12Z,15Z)/18:4 (6Z, 9Z,12Z,15Z)	PC(18:3/18:4)	Glycerophospho- lipids	Glycerophosphocholine
						2-Piperazinecarboxamide	2-PCA	Nitrogen-containing heterocyclic compounds	Piperazine derivatives
	Ethyl 2E,4Z-hexadecadienoate	HDDE-Et	Fatty acid esters	Alkenoate esters		Uncyclized xanthommatin	Uc-Xan	Alkaloids	Ommochromes
Fruit Hosts	Glucose-6-phosphate	G6P	Carbohydrates	Glucose derivatives	Fruit Larvae	UDP-glucose	UDPG	Pyrimidine nucleotides	Pyrimidine nucleotide sugars
	Ethyl 2-hydroxy-3-methylbutanoate	EHMB	Fatty acyls	Hydroxycarboxylate esters		Succinic acid	SA	Carboxylic acid derivatives	Dicarboxylic acids
	2-amino-4-hydroxypteridine	Pte	Pteridine derivatives	Pterin derivatives		Threonyl-histidine	Thr-His	Peptides	Dipeptides
	Isoamyl acetate	IAAc	Fatty acyls	Acetate esters					
	Hyperoside	Hyp	Phenols	Flavone glycosides		Vanillactic_acid	VLA	Phenylpropanoids	Phenylpropionic acids
	Gallic acid	GA	Phenols	Polycyclic phenols		Terephthalic acid	TPA	Carboxylic acid derivatives	Aromatic dicarboxylic acids
	Tartaric acid	TA	Carboxylic acid derivatives	Dicarboxylic acids		Torvoside_A	TDA	Steroids and steroid derivatives	Steroidal saponins
	L-pyrrolidine-2-carboxylic acid	Pro	Carboxylic acid derivatives	Imino acids					

## Data Availability

LC-MS data of some key metabolites have been attached in Supporting Information and also can be found in results of text.

## Supplementary Materials

The following supporting information can be downloaded at: https://www.mdpi.com/article/10.3390/insects17090962/s1.
