# Supplementary material for: Matched Fruit–Larva Metabolomics Identifies Host-Associated Metabolic Signatures After Multi-Generational Laboratory Acclimation in Zeugodacus tau (Tephritidae: Diptera)
Source: Insects. 2026 Sep 16;17(9):962. doi: 10.3390/insects17090962 (PMC13607736; doi:10.3390/insects17090962)
Supplement: Supplementary file 1 [file insects-17-00962-s001.zip › Table S1.pdf]

Table S1: Hosts and larvae samples information form.

**Host species**	**Host Sample Name**	**Host Sample Code**	**Sample Size (mg)**	**Larvae Type**	**Larvae Sample Name**	**Larvae Sample Code**	**Sample Size (mg)**
Cucurbit Hosts	Pumpkin (*Cucurbita moschata*)	NG	534.2	Cucurbit Larvae	Pumpkin Larvae	*Z.tau*-NG	261.2
			544.8				252.8
			559.3				265.4
			502.6				282.3
	Summer squash (*Cucurbita pepo*)	XHL	576.3		Summer squash Larvae	*Z.tau*-XHL	290.8
			518.8				292.5
			543.3				297.8
			551.0				277.5
	Cucumber (*Cucumis sativus*)	HG	574.1		Cucumber Larvae	*Z.tau*-HG	290.8
			599.4				210.5
			555.4				221.5
			506.1				286.6
Fruit Hosts	Banana (*Musa nana*)	XJ	283.0	Fruit Larvae	Banana Larvae	*Z.tau*-XJ	237.8
			283.2				211.7
			293.6				201.6
			287.2				203.9
	Mango (*Mangifera indica*)	MG	590.6		Mango Larvae	*Z.tau*-MG	204.1
			967.3				231.2
			720.5				203.1
			593.7				214.7
	Sweet orange (*Cirtus reticulata*)	JZ	762.4		Orange Larvae	*Z.tau*-JZ	204.9
			732.2				207.3
			641.2				248.4
			667.0				253.0
	Guava (*Psidium guajaval*)	FSL	833.5		Guava Larvae	*Z.tau*-FSL	208.0
			817.1				167.1
			775.0				146.7
			639.6				120.4
	Pitaya (*Hylocereu sundatus*)	HLG	531.8		Pitaya Larvae	*Z.tau*-HLG	223.2
			519.3				284.2
			516.2				243.0
			511.2				245.9
