# Supplementary material for: Matched Fruit–Larva Metabolomics Identifies Host-Associated Metabolic Signatures After Multi-Generational Laboratory Acclimation in Zeugodacus tau (Tephritidae: Diptera)
Source: Insects. 2026 Sep 16;17(9):962. doi: 10.3390/insects17090962 (PMC13607736; doi:10.3390/insects17090962)
Supplement: Supplementary file 1 [file insects-17-00962-s001.zip › Table S2.pdf]

Table S2. Batch-matching information for fruit and larval metabolomic samples.

Batch ID	Host Species	Fruit Quantity	Starting Adult Number	Fruit Sample Code	Larvae Sample Code	Batch Description
Batch-NG-01	Pumpkin *(Cucurbita moschata)*	/	40 (20 females + 20 males)	NG-01	ZNG-01	Same parallel rearing batch
Batch-NG-01	Pumpkin *(Cucurbita moschata)*	/		NG-02	ZNG-02	Same parallel rearing batch
Batch-NG-02	Pumpkin *(Cucurbita moschata)*	/		NG-03	ZNG-03	Same parallel rearing batch
Batch-NG-02	Pumpkin *(Cucurbita moschata)*	/		NG-04	ZNG-04	Same parallel rearing batch
Batch-XHL-01	Summer squash *(Cucurbita pepo)*	/	40 (20 females + 20 males)	XHL-01	ZXHL-01	Same parallel rearing batch
Batch-XHL-01	Summer squash *(Cucurbita pepo)*	/		XHL-02	ZXHL-02	Same parallel rearing batch
Batch-XHL-02	Summer squash *(Cucurbita pepo)*	/		XHL-03	Z.tau-XHL-03	Same parallel rearing batch
Batch-XHL-02	Summer squash *(Cucurbita pepo)*	/		XHL-04	ZXHL-04	Same parallel rearing batch
Batch-HG-01	Cucumber *(Cucumis sativus)*	/	40 (20 females + 20 males)	HG-01	ZHG-01	Same parallel rearing batch
Batch-HG-01	Cucumber *(Cucumis sativus)*	/		HG-02	ZHG-02	Same parallel rearing batch
Batch-HG-02	Cucumber *(Cucumis sativus)*	/		HG-03	ZHG-03	Same parallel rearing batch
Batch-HG-02	Cucumber *(Cucumis sativus)*	/		HG-04	ZHG-04	Same parallel rearing batch
Batch-XJ-01	Banana *(Musa nana)*	/		XJ-01	ZXJ-01	Same parallel

						rearing batch
Batch-XJ-01	Banana *(Musa nana)*	/		XJ-02	ZXJ-02	Same parallel rearing batch
Batch-XJ-02	Banana *(Musa nana)*	/	40 (20 females + 20 males)	XJ-03	ZXJ-03	Same parallel rearing batch
Batch-XJ-02	Banana *(Musa nana)*	/		XJ-04	ZXJ-04	Same parallel rearing batch
Batch-MG-01	Mango *(Mangifera indica)*	/		MG-01	ZMG-01	Same parallel rearing batch
Batch-MG-01	Mango *(Mangifera indica)*	/	40 (20 females + 20 males)	MG-02	ZMG-02	Same parallel rearing batch
Batch-MG-02	Mango *(Mangifera indica)*	/		MG-03	ZMG-03	Same parallel rearing batch
Batch-MG-02	Mango *(Mangifera indica)*	/		MG-04	ZMG-04	Same parallel rearing batch
Batch-JZ-01	Sweet orange *(Citrus reticulata)*	/		JZ-01	ZJZ-01	Same parallel rearing batch
Batch-JZ-01	Sweet orange *(Citrus reticulata)*	/	40 (20 females + 20 males)	JZ-02	ZJZ-02	Same parallel rearing batch
Batch-JZ-02	Sweet orange *(Citrus reticulata)*	/		JZ-03	Z.tau-JZ-03	Same parallel rearing batch
Batch-JZ-02	Sweet orange *(Citrus reticulata)*	/		JZ-04	ZJZ-04	Same parallel rearing batch
Batch-FSL-01	Guava *(Psidium guajava)*	/		FSL-01	ZFSL-01	Same parallel rearing batch
Batch-FSL-01	Guava *(Psidium guajava)*	/	40 (20 females + 20 males)	FSL-02	ZFSL-02	Same parallel rearing batch
Batch-FSL-02	Guava *(Psidium guajava)*	/		FSL-03	ZFSL-03	Same parallel

						rearing batch
Batch-FSL-02	Guava *(Psidium guajava)*	/		FSL-04	ZFSL-04	Same parallel rearing batch
Batch-HLG-01	Pitaya *(Hylocereus undatus)*	/		HLG-01	ZHLG-01	Same parallel rearing batch
Batch-HLG-01	Pitaya *(Hylocereus undatus)*	/	40 (20 females + 20 males)	HLG-02	ZHLG-02	Same parallel rearing batch
Batch-HLG-02	Pitaya *(Hylocereus undatus)*	/		HLG-03	ZHLG-03	Same parallel rearing batch
Batch-HLG-02	Pitaya *(Hylocereus undatus)*	/		HLG-04	ZHLG-04	Same parallel rearing batch

Note: fruit quantity was adjusted according to fruit size and consumption rate in each cage; precise fixed quantity was not recorded.

Note: Fruit and larval samples sharing identical Batch-ID derive from the same parallel rearing batch. Larval replicates were raised on host material from the corresponding batch but were NOT harvested from the exact same individual fruit used for fruit metabolome profiling. There is no strict one-to-one individual-fruit-larva pairing in this experiment.
