# Supplementary material for: Matched Fruit–Larva Metabolomics Identifies Host-Associated Metabolic Signatures After Multi-Generational Laboratory Acclimation in Zeugodacus tau (Tephritidae: Diptera)
Source: Insects. 2026 Sep 16;17(9):962. doi: 10.3390/insects17090962 (PMC13607736; doi:10.3390/insects17090962)
Supplement: Supplementary file 1 [file insects-17-00962-s001.zip › Table S3.pdf]

Table S3: Annotation details of key metabolites from host fruits and *Z. tau* larvae.

Host Group	Abbreviation	KEGG COMPOUND ID	mz	rt	type	level	Larvae Group	Abbreviation	KEGG COMPOUND ID	mz	rt	type	level
								Glu	C00025	148.0603	230.8	POS	Level1
Cucurbit Hosts	GABA	C00334	104.0708	221.7	POS	level1	Cucurbit Larvae	PC(18:3/18:4)	/	776.5206	80.1	POS	Level1
								2-PCA	/	130.0975	275.2	POS	level2
	HDDE-Et		279.234	208.1	NEG	Level2		Uc-Xan	/	441.1056	242.2	NEG	Level2
	G6P	C00092	259.0234	8.7	NEG	level1		UDPG	C00029	565.0487	249.9	NEG	Level1
	EHMB		133.0869	39.5	POS	level1		SA	C00042	117.0192	78.5	NEG	Level1
	Pte	C00715	162.0413	75.0	NEG	level2		Thr-His	/	255.1079	301.2	NEG	level2
Fruit Hosts	IAAc	C12296	131.1067	28.3	NEG	level2	Fruit Larvae						
	Hyp		463.0882	212.9	POS	level2		VLA	/	213.0748	224.8	POS	level2
	GA	C01424	169.0137	143.5	POS	level2		TPA	C06337	165.0266	126.2	NEG	level2
	TA	C00552	151.0971	204.1	POS	Level3		TDA	/	905.5120	123.6	POS	level2
	Pro	C00148	116.0709	189.1	POS	level1							
